# Publications pattern of clinical epilepsy research in Saudi Arabia

**DOI:** 10.17712/nsj.2017.4.20170231

**Published:** 2017-10

**Authors:** Saleh S. Baeesa, Yazid E. Maghrabi, Muad S. Baeesa, Fadi M. Jan, Mohammed M. Jan

**Affiliations:** *From the Division of Neurosurgery (Baeesa S, Maghrabi), from the Department of Pediatrics (Jan M), and from the Faculty of Medicine (Baeesa M, Jan F), King Abdulaziz University, Jeddah, Kingdom of Saudi Arabia*

## Abstract

**Objective:**

To assess the progress in the field clinical epilepsy in Saudi Arabia, by analyzing in depth the research output productivity and publication pattern, and to identify the current situation of epilepsy research and offer solutions.

**Method:**

Literature search strategy was designed to retrieve accessible articles that are related to epilepsy utilizing PubMed, Google Scholar, and Embase. The retrieved articles were analyzed with several parameters, then evaluated using Oxford Center of Evidence Based Medicine level of evidence scale.

**Results:**

Of all identified articles, 90 were conducted in Kingdom of Saudi Arabia and therefore were included. The included articles had a frequency of only 3.5 publications per year, and growth of 24.4% between the periods of 1990-2003 and 2004-2016. Only 13.3% of the articles were related to surgical epilepsy but the majority (86.7%) were related to medical epilepsy. Many articles (53.3%) were level III studies. The most common study design was retrospective studies in 35.6%, and the citations number ranged from 1–289 (Mean=7).

**Conclusion:**

Pattern of publications in clinical epilepsy researches revealed a slow growth rate in the frequency and a lower significance in the quality throughout the past 26 years. Active institutional and national promotion of clinical research is needed to help assess and therefore improve the quality of the provided epilepsy services.

Epilepsy is a common non-communicable chronic disorder that affects the brain of approximately 50 million people worldwide at any age.^1^ It has been estimated that the prevalence of epilepsy in Saudi Arabia is around 6.54/1000, which is comparable to many countries in the region such as Ecuador, USA, Nigeria, China, and India.^2^ Epilepsy poses a broad range of social and psychological difficulties not for the patients themselves, but also for their families.^3^ Improvement of the field of epilepsy, including both surgical and medical aspects, is essential for patients’ wellbeing, and this can be monitored by assessing research output in the field and analyzing such production. Studies of publication patterns in epilepsy are lacking, there are only some scarce studies that addressed such an issue.^4^ Our main objective is to evaluate the improvement of the field epilepsy in Saudi Arabia, by analyzing in depth the research output and publication pattern, and to analyze the current situation of epilepsy research and offer solutions.

## Methods

### Study design and search strategy

This study was carried out between September - December 2016 at king Abdulaziz university hospital, Jeddah, Kingdom of Saudi Arabia. A search strategy was designed to retrieve all articles that are related to either medical or surgical epilepsy. Databases were accessed and the following phrases were utilized: “Search term” AND “Saudi Arabia”. Time interval was restricted to 1/1/1990-31/12/2016. Each article was identified by abstract screening, then inclusion criteria was applied, followed by accessing the full-text to retrieved more data.

### Inclusion/exclusion criteria

Articles in clinical epilepsy, published between January 1990 and December 2016 in English were included. Moreover, only publications with a first author affiliated with a Saudi institution, and a population studied residing in a Saudi institution were included. Also, to be included, the study should be conducted in part in Saudi Arabia. The published article should have a full text available and accessible for further analysis.

Publications published before January 1990, related to the basic neuroscience aspects of epilepsy, study population based in a geographical area outside Saudi Arabia, and articles with no available full text were excluded.

### Information sources

Systemic search was carried out to retrieve each relevant article using both PubMed, Google Scholar, and Embase, by using search terms developed by the research team and related to both surgical and medical epilepsy (**Table 1**). Articles were reviewed by 2 independent reviewers, and graded using Oxford Centre for Evidence-Based Medicine - Levels of Evidence Scale.^5^

**Table 1:** Search terms used in the acquisition of the data of this study.

Terms
Epilepsy surgery, seizure surgery, mesial temporal sclerosis, corpus callosotomy, amygdalo-hippocampectomy, hemispherectomy, multiple subpial transection, vagus nerve stimulation, temporal lobectomy, intractable seizure, seizure, generalized seizure, tonic-clonic, myoclonic, focal seizure, petit-mal, grand-mal, Lennox-Gastaut, Todd’s paralysis, Valproic acid, lamotrigine, Levetiracetam, phenytoin, carbamazepine, West syndrome, electroencephalogram

### Study selection process

After the review process, studies that are published in English, medical or surgical epilepsy related, published between January 1990 and December 2016, with the first author being affiliated with a Saudi institution, are included for final analysis.

### Data items and data collection process

Several parameters were collected from each article such as journal name, impact factor, year of publication, the affiliation of the first author, city, study design, population, citation numbers, study title, database, and corresponding sector. Those parameters were collected in Microsoft Excel spreadsheet.

### Statistical analysis

Microsoft Excel (Microsoft, Redmond, Washington, DC, USA) was used for statistical analysis. Measures of central tendency such as mean and median were used for most parameters, along with parentage. A *p*<0.05 and confidence interval of 95% were considered statistically significant.

## Results

**Figure 1:**
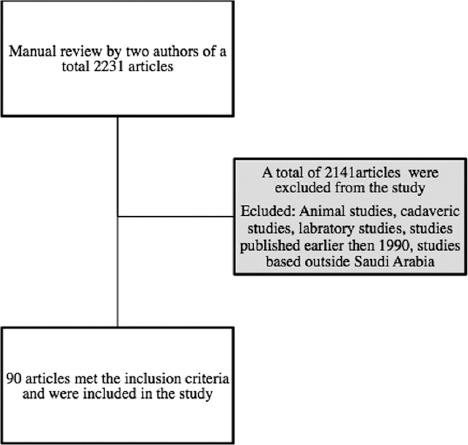
Schematic representation of the review process

Out of 2231 articles identified in our literature search, only 90 met the eligibility criteria of this study. The rest of screened studies were excluded due to the failure to meet the preset inclusion criteria (**Figure 1**). The degree of agreement between the 2 reviewers was very good (Kappa=0.89).

Saudi institutions produced 90 clinical articles over the period of 26 years, with a frequency of 3.5 publications per year, and growth of 24.4% between 1990-2003 and 2004-2016. Twelve (13.3%) of the articles were related to surgical epilepsy, whereas 78 (86.7%) were related to medical epilepsy. Of the 78 (82.6%) articles related to epilepsy Neurology, 42 (58.8%) were related to adult epilepsy neurology and 36 (46.2%) were related to pediatric epilepsy neurology.

Looking at the quality of the included publications, it was found that 48 (53.3%) of the articles represented level III, followed by level IV with 27 (30%), level V 14 (15.6), and level II with 1 (1.1%). In regard impact, citation numbers of all the included articles ranged from 1-289 (median 7) and 15 articles with no citation numbers. **Table 2** represents the 10 most cited clinical epilepsy articles in Saudi Arabia.

**Table 2:** The 10 most cited articles of clinical epilepsy in Saudi Arabia.

Ranks	Articles	Number of Citations
1	Panayiotopoulos CP, Obeid T, Tahan AR. Juvenile myoclonic epilepsy: a 5-year prospective study. ***Epilepsia*** 1994; 35: 285-296.^12^	289
2	Panayiotopoulos CP, Tahan R, Obeid T. Juvenile myoclonic epilepsy: factors of error involved in the diagnosis and treatment. ***Epilepsia*** 1991; 32: 672-676.^15^	135
3	Al Rajeh S, Awada A, Bademosi O, Ogunniyi A. The prevalence of epilepsy and other seizure disorders in an Arab population: a community-based study. ***Seizure*** 2001; 10: 410-414.^2^	76
4	Al-Otaibi FA, Hamani C, Lozano AM. Neuromodulation in epilepsy. ***Neurosurgery*** 2011; 69: 957-979.^16^	46
5	Salih MA, Kabiraj M, Al-Jarallah AS, El Desouki M, Othman S, Palkar VA. Hemiconvulsion-hemiplegia-epilepsy syndrome. A clinical, electroencephalographic and neuroradiological study. ***Childs Nerv Syst*** 1997; 13: 257-263.^17^	45
6	Jan MM, Girvin JP. Seizure semiology: value in identifying seizure origin. ***Can J Neurol Sci*** 2008; 35: 22-30.^18^	41
7	Singh B, Al Shahwan A, Al Deeb SM. Partial seizures presenting as life-threatening apnea. ***Epilepsia*** 1993; 34: 901-903.^19^	38
8	Jan MM, Shaabat AO. Clobazam for the treatment of intractable childhood epilepsy. ***Saudi Med J*** 2000; 21: 622-624.^20^	36
9	Obeid T. Clinical and genetic aspects of juvenile absence epilepsy. ***J Neurol*** 1994; 241: 487-491.^21^	34
10	Yaqub BA. Electroclinical seizures in Lennox-Gastaut syndrome.***Epilepsia*** 1993; 34: 120-127.^22^	31

The most common study design was retrospective studies with 32 (35.6%), then prospective, case reports, and literature review articles; 14 articles each, and comprising 15.6%. Cross-sectional studies 10 (11.1%), Case Series 4 (4.4%), case control 1 (1.1%), and one randomized controlled trial (RCT) (1.1%). The pediatric population was the focus of 36 (40%) of the articles, adults 24 (26.7%), and mixed populations were in 13 (14.4%). In approximately 17 (18.9%) there was no specified population.

Forty-three (47.8%) of publications output was from Riyadh 43 (47.8%), followed by Jeddah 26 (28.9%), Al-Khobar 6 (6.7%), Abha 3 (3.33%), and the remainder of 12 (13.3%) from 9 different cities. (**Table 3**) represent the top 10 Saudi institutions and their contribution to epilepsy literature.

**Table 3:** Top 10 Saudi institutions and their contribution to epilepsy literature.

Centers	n	(%)
King Saud University	22	(24.4)
King Abdulaziz University	16	(17.8)
King Faisal Specialist Hospital and Research Centre- Jeddah	7	(7.8)
Imam Abdulrahman AlFaisal University	6	(6.7)
King Faisal Specialist Hospital and Research Centre- Riyadh	5	(5.6)
King Khalid National Guard Hospital	5	(5.6)
King Fahad Medical City	4	(4.4)
King Khalid University	3	(3.3)
Riyadh Military Hospital	3	(3.3)
Riyadh Armed Forces Hospital	2	(2.2)
Others	14	(18.9)

In regard sectors, the majority of the articles produced by academic institutions 56 (62.2%), governmental institutions 19 (21.1%), and military institutions 15 (16.7%). International collaboration was present only in 7 (7.8%) of articles, whereas there was no international collaboration in 83 (92.2%) of articles. Journals’ impact factor ranged from 0.61-8.17 (median 0.75), 7 articles published in journals with no recorded impact factor. (**Table 4**) show the top 10 journals with their respective number of publications. **Table 5** offers a comparison between epilepsy surgery and epilepsy neurology articles using different variables.

**Table 4:** Top journals with their respective number of publications.

Journals	n	(%)
Neurosciences (Riyadh)	27	(30.0)
Seizure	10	(11.0)
Pediatric Neurology	5	(5.6)
Epilepsy	5	(5.6)
Epilepsy & Behavior	4	(4.4)
Saudi Medical Journal	3	(3.3)
The Canadian Journal of Neurological Sciences	3	(3.3)
Epilepsy Research and Treatment	3	(3.3)
Others	14	(33.3)

**Table 5:** Comparison between surgical vs medical epilepsy articles.

Variables	Surgical epilepsy (n=12)	Medical epilepsy (n=78)
***Level of Evidence (LOE)***		
I	0	0
II	0	1 (1.3)
III	3 (25)	45 (57.7)
IV	4 (33.3)	23 (29.5)
V	5 (41.7)	9 (11.5)
***Citation numbers (CN)***		
Range	2-46	1-289
Median	6	7
Articles with no CN	6	9
***Study design***		
Case-control	0	1 (1.3)
Case reports	4 (33.3)	10 (12.8)
Case series	0	4 (5.1)
Cross-sectional	0	10 (12.8)
Literature review	5 (41.7)	9 (11.5)
Prospective studies	0	14 (17.9)
Randomized controlled studies	0	1 (1.3)
Retrospective studies	3 (25)	29 (37.2)
***Study population***		
Adult	2(16.7)	22 (28.2)
Pediatrics	3(25)	33 (42.3)
Mixed	2(16.7)	11 (14.1)
Not specified	5(41.7)	12 (15.4)
***Top cities***		
1	Riyadh 6 (50)	Riyadh 37 (47.4)
2	Jeddah 3 (25)	Jeddah 23 (29.5)
***Top affiliations***		
1	KFSHRC - Riyadh 5 (41.7)	KSU 22 (28.2)
2	KFSHRC - Jeddah 3 (25)	KAU 16 (20.5)
***Sector***		
Academic	2 (16.2)	54(69.2)
Governmental	10 (83.3)	9(11.5)
Military	0	15(19.2)
***International collaboration***		
Yes	3 (25)	4 (5.1)
No	9 (75)	74 (94.9)
***Journals’ Impact Factor***		
Range	0.66-3.78	0.61-8.17
Median	0.75	0.82
Articles in journals with no IF	5	2
***Top Journals***		
1	Epilepsy Research and Treatment 3 (25)	Neurosciences (Riyadh) 25 (32.1)
2	Neurosciences 2 (16.7)	Seizure 10 (12.8)

KFSHRC - King Faisal Specialist Hospital and Research Centre, KSU - King Saud University, KAU - King Abdulaziz University

## Discussion

Our study documented that there is a lack of literature that addresses publications patterns in epilepsy. These studies are the cornerstone of evaluating the advancement of the field of epilepsy. Looking at Saudi data across the time of 26 years, we find that 90 clinical articles with frequency of 3.5 publications per year, and growth rate of 24.4% between 1990-2003 and 2004-2016, as modest when comparing it with other studies such as the one by Gupta et al^4^ from India, where the output is 1550 paper in only 10 years, with frequency of 155 per year.

**Figure 2:**
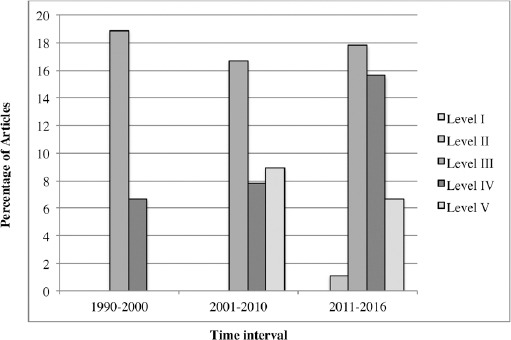
A graphic demonstration of percentage of Saudi epilepsy publications with their respective time interval and level of evidence.

Focusing at the time between 1990-2000, most articles were of level III & IV, with no presence of high-level studies (level I&II). Collectively, Level III & IV in that time stamp constituted 25.6% of the total Saudi research output, with level III constituting the majority (18.9%). Moving toward to the time of 2001-2010, Level III parentage decreased by 2.2%, and level IV improved by 1.1%, which is considered low, and the appearance of level V studies. Finally, comparing the previous results with the interval between 2011-2016, we find the appearance of level II studies, a slight increase in level III frequency to 2001-2010 level, an increase of level IV frequency to 7.8%, and decrease in level V studies by 2.2%. This implies that there is growth in frequency (25.6% in 1990-2000, 33.4% in 2001-2010, 40.1% in 2011-2016), but the growth is slow, with somewhat static LOE quality (**Figure 2**).

Gupta et al^4^ were the first to discuss epilepsy research output restricted to a certain geographical location, namely, India. In their methodology they only used one database; Scoups, as the only database to retrieve articles, with only “Epilepsy” and “India” as keywords. They found that epilepsy research output in India from 2002-2011 consisted of 1550 publications; with average citation number per individual publication was 2.77. Moreover, there was no mention of LOE of the included articles, and citation numbers were the only variables used to assess quality of included publications. The most cited paper in their study received 217 citations.

Rasolabadi and his collogues^6^ followed the footsteps of Gupta et al^4^ and conducted a similar study in Iran.Their methodology was similar to the one used by Gupta et al,^4^ including the use of Scoups as the only database. They included only 702 articles in their study over the period of 14 years with average citations per publication being 4.56. Similar Gupta et al^4^ work; there was also no mention of LOE of included articles. The most cited article included in their study received only 109 citations.

Considering the previously mentioned publications, our obtained results are inferior in terms of frequency being 90 articles in comparison to 1550 in India, and 702 in Iran, even though, we included a wide timeframe; 26 years. On the other hand, our obtained results were superior in terms of average citations number per publication being 14.8, in comparison to 2.77 in India, and 4.56 in Iran. Moreover, the most cited article included in our study received 289, unlike India, which received 217, and Iran, which received 109. Nevertheless, there was no mention of LOE in the previously mentioned studies.

In our opinion, retrieving article from only a single database would result in missing some articles, which in turn lead to giving a wrong estimate of the research output. Moreover, relying only on citation numbers as a sole quality indicator would not be sufficient, and has to be coupled with LOE to serve such a purpose.

When comparing the LOE of the obtained data of this current study, with other studies that dealt with the issue of quality of publications in neurosurgery, orthopedics, and plastic surgery, it has been found that unlike these studies, where all articles are of level IV, most of the articles in epilepsy represent level III.^7^-^11^ Moreover, there was tendency in the field of epilepsy to publish retrospective studies, rather than case reports in the studies mentioned above.^7^-^11^

Looking at the impact of Saudi epilepsy publications on international literature, it appears that impact is modest (citations numbers range 1-289), and only one article by Panayiotopoulos et al,^12^ which has citations number of 289, approaching but not reaching to be a citation classic. Citation classics are defined as the articles that get cited more than 400 times.^13^ A point worth mentioning, Saudi authors tend to publish in Saudi journals, this might be attributed to the issue that the topic of papers would be of interest for physicians residing in Saudi Arabia rather than global community. These journals might have low IF or no IF at all, so the comparison of impact regarding Saudi epilepsy publications must be interpreted with caution.

The field of epilepsy surgery has started in Saudi Arabia in 1998.^14^ Looking at epilepsy surgery publication in Saudi Arabia, we find that it constitutes a very low number being only 12 articles, including literature review articles, in 26 years. These articles are characterized to be produced from centers with advanced capabilities in epilepsy surgery such as King Faisal Specialist Hospital and Research Centre (KFSH&RC). This clarifies many points such that epilepsy surgery in Saudi Arabia is still a new field, with the need of centers with advanced capabilities, and that there are only few epilepsy surgeons in the country.

Epilepsy research in Saudi Arabia faces 2 main problems, the issue of quantity of publications, and their quality. Moreover, Saudi Arabia has an established epilepsy society, which can be the solution to the previously mentioned problems as follow: (1) development of data registry unit that can aid researchers in producing high-quality studies, (2) helping the collaboration of different centers in different parts of the kingdom in conducting RCT,^13^,^14^ (3) developing in collaboration with training centers to develop in-training research fellowships focusing on different aspects of epilepsy,^13^,^14^ (4) finally, offering summer medical students epilepsy research programs, to build an interest in research and epilepsy in young aspiring students.^13^,^14^ In regard epilepsy surgery, the problem is more fundamental involving many aspects; finical, logistic, ethical, and governmental, which is beyond the scope of this paper. Finally, only digital available data was included in this study.

This study has limitations; firstly, the data of this current study did not include other basic sciences types of publications such as animal studies and lab bench studies, as it might of strong study design, good data collection, and solid recommendation that can affect both patient care and publication pattern, regardless of it being of level V-LOE in scales designed only for clinical articles. The factor of bias cannot be eliminated.

We conclude from this review that the publications’ pattern of clinical epilepsy revealed a slow growth rate in the frequency of research with a lower significance in the quality throughout the past 26 years. The current growth of epilepsy centers in Saudi Arabia and post-training epilepsy fellowship program should encourage and promote a larger scale and higher quality research. The active roles of leading institutions should embrace promotion of clinical research to help assess and therefore improve the quality of the provided epilepsy services.
